# Health-related quality of life and treatment satisfaction of patients with cardiovascular disease in Ethiopia

**DOI:** 10.3389/fpubh.2022.972378

**Published:** 2022-10-10

**Authors:** Kebron Tito, Girma Tekle Gebremariam, Kebede Beyene, Beate Sander, Gebremedhin Beedemariam Gebretekle

**Affiliations:** ^1^School of Pharmacy, Addis Ababa University, Addis Ababa, Ethiopia; ^2^Department of Pharmaceutical and Administrative Sciences, University of Health Sciences and Pharmacy in St. Louis, St. Louis, MO, United States; ^3^Institute of Health Policy, Management, and Evaluation, University of Toronto, Toronto, ON, Canada; ^4^Toronto Health Economics and Technology Assessment (THETA) Collaborative, University Health Network, Toronto, ON, Canada; ^5^Institute for Clinical Evaluative Sciences (ICES), Toronto, ON, Canada; ^6^Public Health Ontario, Toronto, ON, Canada

**Keywords:** cardiovascular disease, EQ-5D-5L, Ethiopia, HRQoL, treatment satisfaction

## Abstract

**Purpose:**

Cardiovascular disease is the most prevalent health problem associated with poorer health-related quality of life (HRQoL). We aimed to assess HRQoL and treatment satisfaction of cardiovascular disease patients in Ethiopia.

**Methods:**

A cross-sectional survey was conducted among adults attending the outpatient cardiac clinic at Tikur Anbessa Specialized Hospital from July to September 2021. Patients were recruited consecutively during follow-up visits. Treatment Satisfaction Questionnaire for Medication and European Quality of life questionnaires were used to evaluate treatment satisfaction and HRQoL, respectively. Kruskal-Wallis and Mann-Whitney *U*-tests were used to compare utility weights between patient subgroups. Utility values were computed using disutility weights of the Ethiopian general population derived using a hybrid regression model. Tobit regression modeling was used to explore factors associated with poor HRQoL. Statistical significance was determined at *p* < *0.05*.

**Results:**

A total of 357 patients participated in the study with a mean age of 49.3 ± 17.8 years. The most frequently reported health problems were pain/discomfort (75.4%), followed by mobility (73.4%). The median (interquartile range) European Quality questionnaires five dimensions with five levels utility (EQ-5D-5L) and European Quality of life Visual Analog Scale scores were 0.84 (0.55–0.92) and 70.0 (50.0–85.0), respectively. The highest and lowest mean (standard deviation) treatment satisfaction scores were for the convenience and safety satisfaction dimensions: 87.7 (17.9) and 53.1 (33.5), respectively. Unemployment, older age, previous hospital admission, non-adherence to lifestyle modification, and presence of three or more cardiovascular disease factors were significantly negatively associated with HRQoL.

**Conclusions:**

Overall, the study found that cardiovascular disease had a profound negative effect on HRQoL and patient treatment satisfaction. We suggest that interventions to enhance HRQoL and treatment satisfactions should focus on modifiable associated factors including lifestyle changes and controlling disease progression.

## Introduction

Cardiovascular disease (CVD) is a group of disorders affecting the heart and/or blood vessels (1, 2). It is the leading cause of disability and premature death globally and has significant health and economic burden on patients, families, and healthcare systems (1). According to 2022 American Heart Association report, an estimated 19 million CVD-related deaths occurred globally, with three-quarters of these deaths occurring in low- and middle-income countries (LMICs) (3). In Ethiopia, the reported prevalence of CVD ranges from 1 to 24% with a pooled prevalence of 5%, and significantly contributes to increasing healthcare costs (4, 5). CVD and its treatments pose significant burden on patients' health-related quality of life (HRQoL) and can affect their ability to function (6, 7).

HRQoL is a patient-reported outcome measure that evaluates how a patient's health status is affected by disease, complications, and therapy (8, 9). It can provide information about a person's overall health state that is linked with physical, social, and mental health as well as their respective impact on health status of the patient that is affected by individual's beliefs, perceptions, experiences, and expectations (10, 11). Thus, HRQoL is an important metric to assess the impact of health interventions and is a useful input for valuing health outcomes in economic evaluations. Likewise, treatment satisfaction is a patient-reported outcome measure that considers the patient's perspective of medical treatment and healthcare services (12) and can be used to predict adherence and persistence of medication use over time (13, 14).

To improve patient outcomes, patient-reported problems must be addressed alongside cardiovascular pharmacotherapy. Previous studies showed that a more active involvement of patient's in disease management can substantially enhance HRQoL (15, 16). In LMICs, lower socioeconomic status, low access and high cost of medications could negatively affect HRQoL and treatment satisfaction. On the other hand, adequate physical activity and younger age were associated with better HRQoL (17). Similarly, patients with controlled blood pressure have better overall satisfaction with their medications and HRQoL. Patients who had no adverse events and experienced less anxiety and depression had significantly higher overall treatment satisfaction and better HRQoL (18–21).

Several generic and disease-specific tools have been developed for measuring HRQoL of patients with CVD (22–25). The European Quality of life five-dimension-5 level scale questionnaire (EQ-5D-5L) is a widely used generic, preference-based multi-attribute utility instrument to quantify the global burden of disease in clinical practice and aids health technology assessment in many jurisdictions (26, 27). The tool measures the health state of the patients by generating a single summary utility value, which reflects how good or bad a health state is based on the preferences of the general population of a country (28). The utility weight generated is an important measure to quantify the impact of disease, measure potential benefits and harms of different interventions; and inform resource allocation (29). However, data on HRQoL and treatment satisfaction of patients with CVD in Ethiopia remains limited. This study aimed to assess HRQoL and its associated factors in a sample of patients with CVD at a tertiary care teaching hospital in Ethiopia. The findings could aid in the development of tailored interventions that improve patient health outcomes.

## Methods

### Study design and setting

An interview-based cross-sectional survey was conducted from July to September 2021 among patients attending an outpatient cardiac clinic at Tikur Anbessa Specialized Hospital in Addis Ababa, Ethiopia. The hospital is the largest tertiary care teaching hospital in the country with over 800 beds and serves over half a million patients per year. Of these, approximately 5,500 patients attend the cardiac clinic annually.

### Patient recruitment and data collection procedure

All patients visiting the cardiac clinic comprised the source population while those who fulfilled the eligibility criteria during the data collection period comprised the study population. The sample size was estimated based on a single population proportion formula (30), considering a *Z*-value of 1.96 with a 95% level of confidence and a 5% margin of error. The prevalence (P) for sample size estimation was based on a previous study conducted in Ethiopia using the World Health Organization Quality of Life questionnaire (WHOQoL-BREF) instrument (15), in which 45.6% of patients with heart failure rated their overall HRQoL as poor. We applied the finite population correction factor formula (31) for the source population of 5,500 CVD patients, and recruited a total of 360 eligible patients using a consecutive sampling method. Patients were eligible if they were 18 years of age or older and diagnosed with CVD. We excluded patients under the age of 18 years, unwilling to participate, and those who had severe cognitive/mental problems as confirmed by the treating physician.

Three clinical pharmacists conducted face-to-face interviews with the patients during their follow-up clinic visit. All data collectors were trained to ensure uniformity and reduce inter-observer bias in data collection. The purpose and procedure of the study were explained to all study participants before data collection. Written informed consent was obtained from all study participants, and personal identifiers were not collected. During the data collection process; data collectors clarified queries raised by patients. Information on sociodemographic characteristics such as sex, age, marital status, occupation, level of education, alcohol habit, smoking status, and lifestyle-related factors were collected through face-to-face interviews. Chart review was undertaken to gather information on clinical characteristics of the patients including duration of CVD, complications, non-cardiac comorbidities, and the number of medications taken. We collected patients' treatment satisfaction and HRQoL using the Treatment Satisfaction Questionnaire for Medication (TSQM) and EQ-5D-5L instruments, respectively. Patients' information regarding medication adherence was collected using Morisky Green-Levine (MGL) questionnaire.

### Instruments

#### EQ-5D-5L

EQ-5D-5L is a generic instrument that consists of five dimensions with five levels, of which four are physical dimensions and one is psychological dimension (28, 32). Validated Amharic version of the EQ-5D-5L was used for the interview. The instrument has two parts: the EQ-5D-5L descriptive system short questionnaires and European Quality Visual Analog Scale (EQ-VAS). In the first part of the instrument, patients select the statement most reflective of their health state in the descriptive system that has five dimensions (mobility, self-care, usual activities, pain/discomfort, and anxiety/depression). Under each dimension, there are five levels (no problem, slight problem, moderate problem, severe problem, and extreme problem) which represent the severity of problems. We asked participants to choose one level that reflects their health state on the interview date for each of the five dimensions. The EQ-VAS is used for subjective assessment of one's current health state from the patient's perspective. Using this scale, each interviewed patient self-rated his/her health status on a vertical scale that ranges from zero (the worst health one can imagine) to 100 (the best health one can imagine).

#### TSQM

TSQM is a 14-item instrument used to evaluate treatment satisfaction with medication over the past 2–3 weeks, or since the patient's last medication use (33). The TSQM has been translated to Amharic language, the national language used in Ethiopia, using forward and backward translation technique. It has four dimensions: effectiveness (three items), safety (five items), convenience (three items), and global satisfaction (three items). Item scores are summed to give four dimension scores, which are in turn transformed to a scale of zero (extremely dissatisfied) to 100 (extremely satisfied) (34). The TSMQ have also been utilized in previous studies in Ethiopia to assess satisfaction with treatment.

### MGL

We used the four-item Morisky Green-Levine (MGL) questionnaire to assess medication adherence. The tool results in a score ranging from 0 to 4. A dichotomous definition of adherence based on MGL used with “0” indicating perfect adherence and “1” indicating some level of non-adherence (35).

### Statistical analysis

Descriptive statistics such as mean and percentage are used to summarize patients' demographic and clinical characteristics. Differences in percentages of reported problems with patients' characteristics were tested using the χ^2^-test. The level of severity for each dimension of the EQ-5D-5L descriptive system was reported using 5 levels to determine the proportion of health problems reported in different subgroups (1 = no problem to 5 = extreme problem). The EQ-5D-5L utility score were computed using disutility coefficients (decrement in utility) obtained from the Ethiopian general population using a hybrid regression model (36). For each of the five health dimension [mobility (mo), self-care (sc), usual activities (ua), pain/discomfort (pd), and anxiety/depression (ad)]; we set four incremental dummies (a total of 20 parameters) by considering “1 = no problem” as a reference and representing the utility decrement when moving from lower level to the next higher level (e.g., moving from *sc1* to *sc2* resulted in a decrease in utility of 0.0235125). The utility value for CVD patients was computed using Equation (1). As the EQ-5D-5L utility and EQ-VAS scores were non-normally distributed *(Shapiro-Wilk test, p* < *0.05)*, we present median [interquartile range (IQR)] scores. We applied Kruskal-Wallis and Mann-Whitney U tests to determine the differences in the EQ-5D-5L utility scores and EQ-VAS among subgroups of patients. A multivariate Tobit regression model was used to examine the predictors of HRQoL. Some covariates, i.e., smoking status and alcohol consumption habits, were not entered into regression models due to a smaller number of responses. We censored the utility score at one and the EQ-VAS score at 100. In addition, Pearson's correlation coefficient was used to assess correlation between EQ-VAS scores, EQ-5D-5L utility index, and TSQM dimension scores. To distinguish higher treatment satisfaction from lower satisfaction in each dimension; we dichotomized the mean TSQM score into ≥75 (higher treatment satisfaction) and <75 (lower treatment satisfaction) (37). Statistical analyses were performed using STATA Version 14. All statistical tests were performed using a level of significance of *p*-value < 0.05.

***Utility value* =** *1-(mo2* * *0.0337341* + *mo3* * *0.0644715* + *mo4* * *0.2276493* + *mo5* * *0.3598963)* + *(sc2* * *0.0235125* + *sc3* * *0.0394815* + *sc4* * *0.1419238* + *sc5* * *0.2223553)* + *(ua2* * *0.0323013* + *ua3* * *0.0482993* + *ua4* * *0.1573934* + *ua5* * *0.2721253)* + *(pd2* * *0.0360808* + *pd3* * *0.0515949* + *pd4* * *0.2703189* + *pd5* * *0.4063984)* + *(ad2* * *0.0258862* + *ad3* * *0.0848133* + *ad4* * *0.2987388* + *ad5* * *0.4577938)* (1)

## Results

### Sociodemographic characteristics

A total of 360 patients were approached to take part in the study of which 357 (99.2%) consented and completed the study questionnaires. The majority of the study participants were females (65.8%), married (57.4%), unemployed (79.6%), and 18–60 years old (69.7%; mean age = 49.3 ± 17.8 years). One in five participants were illiterate, approximately three-fourths of participants were living in Addis Ababa and most participants (60.2%) had health insurance coverage. Detailed sociodemographic patient characteristics are presented in Table 1.

**Table 1:** Sociodemographic characteristics of patients with cardiovascular disease.

**Variables**	***N* (%)**
**Age category (in years)**
18–60	249 (69.7)
>60	108 (30.3)
**Sex**
Male	122 (34.2)
Female	235 (65.8)
**Marital status**
Single	84 (23.5)
Married	205 (57.4)
Divorced	26 (7.3)
Widow	42 (11.8)
**Educational status**
Unable to read and write	72 (20.2)
Primary education	105 (29.4)
Secondary education	113 (31.7)
Post-secondary education	67 (18.8)
**Employment status**
Employed	73 (20.4)
Unemployed	284 (79.6)
**Residence**
Addis Ababa	263 (73.7)
Out of Addis Ababa	94 (26.3)
**Health insurance usage**
Yes	215 (60.2)
No	142 (39.8)

### Clinical characteristics of patients with CVD

The majority (72.8%) of participants had CVD for 5 years or more with a mean duration of 9.57 ± 8.59 years. Three hundred (84%) patients were adherent to lifestyle modifications; most (60.3%) used dietary lifestyle modification (Table 2). Nearly all patients (98.3%) were non-smokers, and 332 (93%) had no alcohol consumption habits within 1 month prior to the interview date. One in five (20.4%) patients had a history of hospital admission in the past year, and the majority (87.7%) of patients had more than two CVDs. Almost half of the patients (45.1%) had non-cardiac comorbidities. Most patients (65.3%) were taking more than five medications, and 204 (57.1%) were adherent to their medications. Unavailability of medications (37.5%) and forgetfulness (29%) were the main reasons to medication non-adherence.

**Table 2:** Clinical characteristics of patients with cardiovascular disease.

**Variables**	***N* (%)**
**Duration of CVD (in years)**
<5 years	97 (27.2)
5–10 years	125 (35.0)
>10 years	135 (37.8)
**Use of lifestyle modifications**
Yes	300 (84.0)
No	57 (16.0)
**Type of lifestyle modifications used**
Diet	181 (60.3)
Exercise	17 (5.7)
Both	102 (34.0)
**Smoking habit**
Yes	5 (1.4)
No	351 (98.3)
**Alcohol use**
Yes	25 (7.0)
No	332 (93.0)
**Previous hospital admission**
Yes	73 (20.4)
No	284 (79.6)
**Non-cardiac comorbidities**
Yes	161 (45.1)
No	196 (54.9)
**Number of CVD**
<3 conditions	313 (87.7)
≥3 conditions	44 (12.3)
**Number of medications**
<5	124 (34.7)
≥5	233 (65.3)
**Adherent to medications**
Yes	204 (57.1)
No	153 (42.9)
**Reasons for non-adherence**
Forgetfulness	49 (32.1)
Unavailability	57 (37.3)
Fear of side effects	7 (4.5)
Others^*^	40 (26.1)

* Lack of transportation, felt well, no belief in effectiveness of medications, got worsen, not afford the cost.

CVD, Cardiovascular disease.

### Distribution of EQ-5D-5L descriptive dimensions

**Figure 1:**
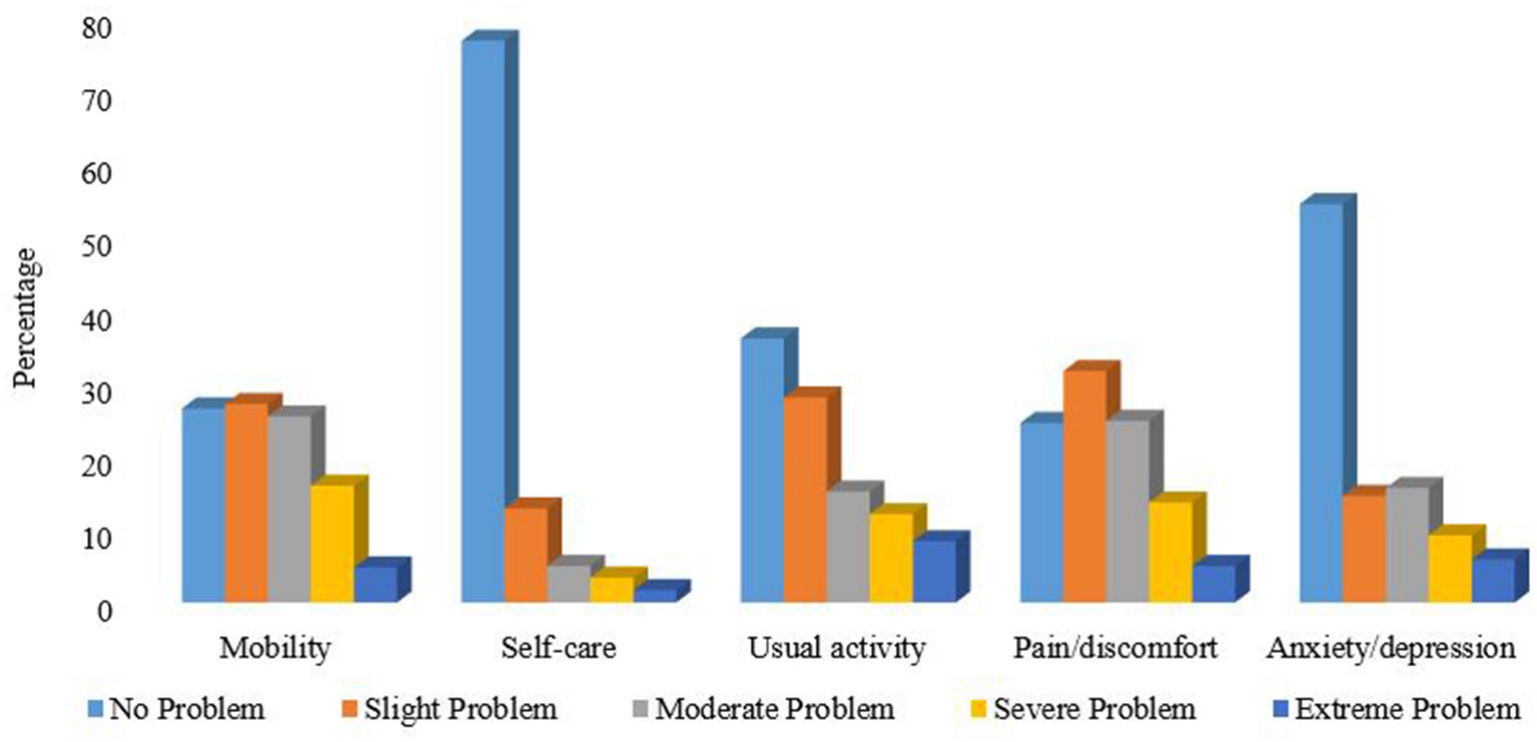
Self-reported health problems using the EQ-5D-5L descriptive dimensions in patients with Cardiovascular disease.

Patients' self-reported health status for the five dimensions of EQ-5D-5L is presented in Figure 1. The most frequent health problems reported were “pain/discomfort” (75.4%, all levels) followed by “mobility” (73.4%, all levels), while the least health problem was “self-care” (23%, all levels). Thirty-seven (10.4%) participants reported “no problem” or a “perfect health state” (11,111) in the EQ-5D-5L descriptive dimension, whereas only 30 (8.4%) reported the “best health state” (100) in the EQ-VAS. The frequencies of any problem reported with all dimensions were significantly different across patient characteristics such as sex, educational status, age, non-cardiac comorbidities, types of lifestyle modifications, previous hospitalization history, medication adherence status as well as the different dimensions of treatment satisfaction. Patients with higher treatment satisfaction (≥75) had lower problem levels in EQ-5D-5L dimensions (mobility, activities, self-care, pain/discomfort, and anxiety/depression) Supplementary File 1.

### EQ-5D-5L index value and EQ-VAS score

The median (IQR) EQ-5D-5L index and EQ-VAS scores were 0.84 (0.55–0.92) and 70 (50–85), respectively. Whereas, the mean (SD) EQ-5D-5L index and EQ-VAS scores were 0.69 (0.34) and 68.7 (21.6), respectively. The median EQ-5D-5L index and EQ-VAS scores were significantly higher in patients with higher educational status compared to illiterate *(p* < *0.05)*. There were significant differences in both EQ-5D-5L utility value and EQ-VAS scores among patients with previous hospital admission history compared to those without [(0.67 vs. 0.86 and (65 vs. 75)], respectively. Significant positive statistical correlations were observed with both median scores of EQ-5D-5L utility and EQ-VAS, and adherence to different types of lifestyle modifications. Conversely, no significant correlation was observed between EQ-5D-5L utility value and EQ-VAS scores, and medication adherence status, number of medications used, non-cardiac comorbidities, and number of cardiac conditions. A significant difference in EQ-5D-5L utility and EQ-VAS scores were found between all satisfaction dimensions score. Patients with higher treatment satisfaction (>75) had higher EQ-5D-5L utility index and EQ-VAS scores (Table 3).

**Table 3:** Median (IQR) differences of EQ-5D-5L utility and EQ-VAS scores with patient characteristics.

**Variables**	**EQ-5D-5L index median score (IQR)**	**Mean rank**	***p*-value**	**EQ-VAS median score (IQR)**	**Mean rank**	***p*-value**
**Sex**	
Male	0.70 (0.57–0.94)	190.6	0.127	75 (50.0–85.0)	176.9	0.784
Female	0.82 (0.52–0.91)	173.0		70 (50.0–85.0)	180.1	
**Age category**	
<60	0.84 (0.58–0.92)	181.1	0.552	70 (50.0–85.0)	180.0	0.500
≥60	0.82 (0.50–0.92)	174.1		75 (50.0–85.0)	176.9	
**Marital status**	
Single	0.82 (0.36–0.90)	164.3	0.195	70 (50.0–88.8)	171.8	0.240
Divorced	0.88 (0.61–0.92)	191.1		66 (48.8–81.3)	161.9	
Married	0.86 (0.58–0.93)	187.2		75 (60.0–85.0)	188.1	
Widow	0.77 (0.45–0.92)	160.8		65 (50.0–80.0)	159.8	
**Employment status**	
Employed	0.86 (0.64–0.93)	194.7	0.114	75 (60.0–90.0)	198.8	0.068
Unemployed	0.82 (0.51–0.92)	166.7		70 (50.0–85.0)	173.9	
**Education status**	
Unable to read and write	0.72 (0.49–0.89)	167.5	**0.001**	85 (47.5–77.5)	178.5	**0.009**
Primary education	0.74 (0.42–0.90)	179.2		70 (50.0–80.0)	177.4	
Secondary education	0.74 (0.42–0.90)	178.3		75 (60.0–85.5)	175.8	
Higher education	0.88 (0.59–0.94)	192.3		78 (63.8–86.3)	187.4	
**Residence**	
Addis Ababa	0.86 (0.58–0.92)	187.6	**0.008**	75 (55.0–85.0)	184.9	0.086
Other	0.69 (0.44–0.90)	155.0		70 (50.0–80.0)	163.4	
**Health insurance usage**	
Yes	0.82 (0.51–0.92)	170.0	**0.042**	70 (50.0–85.0)	171.5	0.088
No	0.86 (0.58–0.94)	192.7		75 (58.8–85.0)	190.4	
**Lifestyle modification**	
Yes	0.85 (0.59–0.92)	185.6	**0.006**	75 (55.0–85.0)	185.7	**0.005**
No	0.72 (0.25–0.90)	144.3		60 (50.0–75.0)	144.0	
**Lifestyle modification type**	
Diet	0.72 (0.47–0.90)	126.5	**0.000**	70 (50.0–80.0)	130.6	**0.000**
Exercise	0.87 (0.83–0.93)	178.5		80 (67.5–90.0)	180.1	
Both	0.90 (0.80–0.96)	188.5		80 (70.0–90.0)	180.8	
**Smoking habit**	
Yes	0.85 (0.59–0.92)	225.8	0.300	75 (55.0–85.0)	140.7	0.406
No	0.72 (0.25–0.89)	177.8		60 (50.0–75.0)	179.0	
**Alcohol use**	
Yes	0.90 (0.72–1.00)	219.5	**0.042**	75 (62.5–90.0)	196.7	0.371
No	0.84 (0.54–0.92)	176.0		70 (50.0–85.0)	177.7	
**History of hospital admission**	
Yes	0.67 (0.34–0.88)	138.1	**0.000**	65 (45.0–80.0)	150.3	**0.008**
No	0.86 (0.59–0.93)	189.5		75 (56.3–85.0)	186.4	
**Time since CVD diagnosis**	
<5 years	0.84 (0.39–0.92)	174.9	0.586	70 (50.0–86.0)	176.6	0.944
5–10 years	0.82 (0.53–0.92)	174.4		75 (50.5–80.0)	181.3	
>10 years	0.86 (0.60–0.92)	186.2		75 (50.0–85.0)	178.6	
**Number of cardiac conditions**	
<3 condition	0.85 (0.59–0.92)	178.6	0.160	70 (50.0–85.0)	177.0	0.37
≥3 conditions	0.87 (0.58–0.92)	183.6		78 (53.7–90.0)	199.4	
**Non-cardiac comorbidities**	
Yes	0.85 (0.57–0.92)	180.7	0.730	70 (50.0–85.0)	178.2	0.87
No	0.84 (0.54–0.92)	176.9		75 (50.0–82.5)	180.0	
**Number of medications**	
<5	0.85 (0.58–0.92)	183.3	0.131	70 (50.0–80.0)	183.4	0.63
≥5	0.74 (0.58–0.93)	163.0		70 (50.0–85.0)	173.9	
**Adherence to medications**	
Yes	0.84 (0.56–0.92)	178.2	0.960	75 (55.0–85.0)	181.5	0.456
No	0.84 (0.51–0.92)	177.7		70 (50.0–80.0)	173.3	
**Effectiveness dimension**	
<75	0.67 (0.32–0.90)	143.4	**0.001**	60 (50.0–80.0)	140.0	0.001
≥75	0.87 (0.66–0.92)	195.5		75 (60.0–90.0)	197.1	
**Safety dimension**	
<75	0.87 (0.61–0.92)	53.7	**0.005**	70 (55.0–85.0)	53.4	**0.008**
≥75	0.49 (0.48–0.87)	36.5		53 (44.0–72.5)	37.1	
**Convenience dimension**	
<75	0.65 (0.29–0.89)	138.8	**0.001**	60 (50.0–80.0)	141.8	**0.001**
≥75	0.86 (0.60–0.92)	188.0		75 (60.0–85.0)	187.3	
**Overall satisfaction dimension**	
<75	0.68 (0.47–0.90)	137.0	**0.001**	70 (50.0–80.0)	137.6	**0.001**
≥75	0.88 (0.97–0.94)	194.0		75 (60.0–90.0)	193.8	

CVD, Cardiovascular disease; EQ-5D-5L, EuroQoL life questionnaires five dimensions with five levels; EQ-VAS, EuroQoL Visual Analog scale; IQR, Interquartile range. Bold text indicates a significant statistical association.

### Treatment satisfaction and its correlation with HRQoL

The mean (SD) treatment satisfaction scores for convenience and overall satisfaction were 87.7 (17.9) and 78.9 (27.7), respectively. On the other hand, the effectiveness and safety satisfaction dimensions (experience of patient encountered side effects) measured over the previous 2–3 weeks were found to be 73.8 (36.7) and 53.1 (33.5), respectively. There were significant modest positive correlations between effectiveness, convenience, and overall treatment satisfaction dimensions with EQ-5D-5L and EQ-VAS scores. The Pearson's correlation coefficient between mean scores of effectiveness and the EQ-5D-5L score was 0.32 while the correlation between the convenience dimension and EQ-5D-5L score was 0.26. Safety satisfaction dimension showed a negative correlation with the EQ-5D-5L index (correlation coefficient = −0.37) and EQ-VAS score (correlation coefficient = −0.34) (Table 4).

**Table 4:** Pearson correlation coefficient between treatment satisfaction and HRQoL.

**Satisfaction dimension**	**Number of items**	**Mean (SD)**	**EQ-5D-5L score**	**EQ-VAS score**
Effectiveness	3	73.8 (36.8)	0.32	0.28
Convenience	3	87.7 (17.9)	0.26	0.22
Safety	5	53.1 (33.5)	−0.37	−0.34
Overall satisfaction	3	78.9 (27.7)	0.26	0.27

EQ-5D-5L, EuroQoL life questionnaires five dimensions with five levels; EQ-VAS, EuroQoL Visual Analog scale; HRQoL, Health-related quality of life.

### Factors associated with health-related quality of life

The multivariable Tobit regression model (Table 5) showed that older age (β = −0.033, 95%CI = −0.151, 0.005), being unemployed (β = −0.087, 95%CI = −0.174, −0.001), previous hospital admission history (β = −0.165, 95%CI = −0.249, −0.082), and presence of three or more cardiovascular diseases (β = −0.103, 95%CI = −0.205, −0.001) were significantly negatively associated with the EQ-5D-5L utility index. Conversely, overall treatment satisfaction dimension (β = 0.003, 95%CI = 0.001, 0.004) and using lifestyle modification (β = 0.164; 95%CI = 0.255, 0.072) were significantly positively associated with EQ-5D-5L utility. Similarly, overall treatment satisfaction dimension (β = 0.002, 95%CI = 0.001, 0.003) and lifestyle modifications (β = 0.086, 95%CI = 0.147, 0.026) were positively associated with EQ-VAS score while previous hospitalization history was negatively associated with EQ-VAS score. Marital status, sex, education status, insurance usage, non-cardiac comorbidities, years since CVD diagnosis, and polypharmacy were not significantly associated with either the EQ-5D-5L index or EQ-VAS scores.

**Table 5:** Predictors of HRQoL in patients with cardiovascular disease.

**Variables**	**EQ-5D-5L index score**		**EQ-VAS score**	
	**β-Coeff. (95 % CI)**	***p-*value**	**β-Coeff. (95 % CI)**	***p-*value**
Female	0.011 (−0.063, 0.085)	0.778	−0.027 (−0.077, 3.33)	0.756
**Level of education (ref** **=** **illiterate)**	
Primary	−0.085 (−0.182, 0.011)	0.072	−0.011 (−0.075, 0.054)	0.746
Secondary and higher	−0.080 (−0.167, 0.007)	0.082	0.012 (−0.070, 0.046)	0.686
**Marital status (ref** **=** **unmarried)**	
Married	−0.048 (−0.117, 0.022)	0.179	−0.034 (−0.081, 0.012)	0.148
**Age (Ref** **=** ** <60 years)**	
≥60 years	−0.033 (−0.151,0.005)	0.027^*^	−0.036 (−0.085, 0.013)	0.144
**Alcohol use (ref** **=** **no)**	
Yes	−0.105 (−0.239, 0.028)	0.120	−0.059 (−0.149, 0.721)	0.191
**Lifestyle modification (ref** **=** **no)**	
Yes	0.164 (0.255, 0.072)	0.001^*^	0.086 (0.147, 0.026)	0.006^*^
**Insurance usage (ref** **=** **no)**	
Yes	0.048 (−0.023, 0.118)	0.183	0.033 (−0.013, 0.080)	0.161
**Employment status (ref** **=** **employed)**	
Non-employed	−0.087 (−0.174, −0.001)	0.049^*^	−0.055 (−0.113, 0.001)	0.056
**History of previous admission (ref** **=** **no)**	
Yes	−0.165 (−0.249, −0.082)	0.001^*^	−0.081 (−0.137, −0.025)	0.005^*^
Years since CVD diagnosis	−0.003 (−0.001, −0.007)	0.191	−0.001 (−0.002, 0.003)	0.663
**Non-cardiac comorbidity (ref** **=** **no)**	
Yes	−0.037 (−0.110, 0.037)	0.322	−0.012 (−0.059, −0.035)	0.614
**Number of CVD (ref** **=** ** <3 conditions)**	
≥3 conditions	−0.103 (−0.205, −0.001)	0.049^*^	−0.025 (−0.093, 0.044)	0.478
**Number of medication (ref** **=** ** <5)**	
>5 medications	−0.010 (−0.083, 0.064)	0.791	−0.028 (−0.077, 0.021)	0.269
**Overall satisfaction**	0.003 (0.001, 0.004)	0.001^*^	0.002 (0.001, 0.003)	0.001^*^

* p-value < 0.05.

CI, Confidence interval; CVD, Cardiovascular disease; EQ-5D-5L, EuroQoL life questionnaires five dimensions with five levels; EQ-VAS, EuroQoL Visual Analog scale; Ref, Reference. Bold text indicates a significant statistical association.

## Discussion

The symptoms and complications of CVD as well as its treatment have a significant impact on patients' HRQoL (6, 7, 19, 37, 38). As a result, HRQoL measures are now widely used to assess disease burden and treatment outcomes in both clinical practice and research. Thus, this study aimed to assess the HRQoL and treatment satisfaction of patients with CVD; compare EQ-5D-5L index and EQ-VAS scores across different levels of patient characteristics, and identify factors associated with HRQoL. Consistent with the previous literature (18, 19, 39, 40), our findings demonstrated that CVD diagnosis was significantly associated with impaired HRQoL. The mean EQ-5D-5L utility value in this study was 0.69, which is lower than the mean utility value of 0.92 for the Ethiopian general population (36). The most frequently reported health problems were pain/discomfort followed by mobility. Further, our study identified factors associated with HRQoL that could be targeted by interventions to improve patients' HRQoL and reduce burden of the disease.

A perfect health state (11,111) was reported only in 37 (10.4%) patients in the descriptive dimension of EQ-5D-5L which is much lower than reported in a Chinese study (55.6%) (39). Similar to previous studies (18, 37, 40), we found that pain/discomfort was the most affected dimension (75.4%). This indicates that CVD could have a detrimental impact on the physical component of patient's HRQoL; particularly due to frequent use of injectable medications, cardiovascular complications such as stroke, and general disease progression (19, 37). The mobility dimension was the second most frequently reported health problem, which could be due to the inclusion of patients with stroke and heart failure in this study. These conditions may cause significant difficulty in climbing stairs and walking. The EQ-5D-5L usual activity dimension was the third most frequently reported problem and this might be due to cardiovascular complications that might have contributed to a decline in daily routine activities of patients. Overall, our findings highlight the importance of paying attention to patients' physical functioning by fostering comprehensive lifestyle changes with physical activity, controlling CVD complications, and non-cardiac comorbidities. Additionally, HRQoL should be part of routine patient assessment and be an integral part of CVD management beyond pharmacotherapy.

The mean EQ-5D-5L utility value of our finding was 0.69, which is lower than that reported by studies from China (0.85), European countries (0.78), Vietnam (0.82), and Hong Kong (0.889) (18, 41–43). The mean EQ-VAS score in our study was 68.7; this is comparable with Henan et al.'s finding among Chinese patients, which was 69.44 (40). We infer from these results that CVD is associated with impaired HRQoL. These differences in utility values could be due to variations in patients' profiles, sociocultural beliefs, and types of CVD across study settings as well as access to medical care and differences in EQ-5D-5L tariffs utilized.

Similar to previous studies (26, 38, 44), we found statistically significant differences in EQ-5D-5L index and EQ-VAS scores among participants with different levels of education, lifestyle modification use, social habits, previous hospital admission, and TSQM dimensions score. Our findings showed that higher treatment satisfaction (TSQM overall score ≥75) was associated with improved HRQoL, which is consistent with previous findings (44). Our findings confirmed that improving treatment satisfaction and improving patients' safety by reducing medication side effects could improve health outcomes. Our findings may help in designing intervention strategies for CVD patients through identifying modifiable factors associated with poorer HRQoL. The modest correlations between TQSM, EQ-5D-5L and EQ-VAS scores could be due to different constructs of the scales. TSQM focuses more on patient judgment about treatment-related experiences and healthcare services, while the EQ-5D-5L dimensions focus more on physical functioning for a health state (12, 44).

The present study identified unemployment, older age, previous hospital admission, non-adherence to lifestyle changes, presence of three or more CVD, and lower overall satisfaction as significant negative predictors of HRQoL. The decline of HRQoL with older individuals could be attributed to increased deterioration of physical functioning, which might increase CVD progression and non-cardiac comorbidities leading to reduced overall HRQoL. Likewise, lower HRQoL among previously hospitalized patients could be explained by hospital admission being mainly due to progression and worsening of the disease conditions (45). Consistent with previous research (37, 38, 41), our findings illustrate that the use of lifestyle changes is positively associated with better HRQoL, and those patients who used physical activity had significantly better HRQoL than patients who did not. Our study also demonstrated that the presence of more than three CVD was an independent predictor of impaired HRQoL. Thus, our findings indicate the importance of regular physical activity as well as reducing the progression of disease in the patients (37). Further, our finding showed that higher overall treatment satisfaction was significantly associated with better HRQoL, demonstrating the importance to focus on increasing patients' treatment satisfaction by ameliorating medication side effects. Therefore, healthcare providers should evaluate patient satisfaction with treatment during routine follow-up.

There are some limitations to this study. First, since our study was a cross-sectional survey, it is not possible to demonstrate causal relationships between associated factors and HRQoL. Second, patients were recruited from one tertiary hospital, and thus, our conclusions cannot be generalized to CVD patients in Ethiopia. Despite these limitations, this was the first study assessing the association between HRQoL and treatment satisfaction in patients with CVD in Ethiopia. Thus, our findings have broad implications for enhancing HRQoL of patients with CVD by identifying modifiable factors for poorer HRQoL that could inform context-specific interventions in resource limited settings. In addition, the utility weight generated is important measure that helps to conduct economic evaluation in the future.

## Conclusions

Our study found that CVD diagnosis had a profound negative effect on HRQoL and patient treatment satisfaction. Most patients reported problems in the pain/discomfort and mobility physical dimensions of the EQ-5D-5L. The median EQ-5D-5L utility and EQ-VAS scores were found to be 0.84 and 70, respectively. Older age, previous hospital admission, non-adherence to lifestyle modifications, and presence of three or more CVD factors were significantly negatively associated with HRQoL. Therefore, future intervention efforts aimed at improving HRQoL and treatment satisfaction in CVD patients should be designed with a focus on modifiable factors such as controlling progression of CVD and promoting lifestyle change. Further, the utility values derived in our study could support economic evaluations to identify cost-effective interventions that can improve health outcomes for patients with CVD in the future.

## Data availability statement

The original contributions presented in the study are included in the article/Supplementary material, further inquiries can be directed to the corresponding authors.

## Ethics statement

The study received ethics approval from the Ethics Review Board of the School of Pharmacy, Addis Ababa University (Protocol#:ERB/SOP/296/13/2021). The patients/participants provided their written informed consent to participate in this study.

## Author contributions

KT, GTG, KB, and GBG contributed to the study concept and design. KT and GTG took the lead in data collection and analysis, in results interpretation, and drafting the first manuscript. KB, BS, and GBG contributed towards data analysis, result interpretation, and revising the manuscript for publication. All authors read and approved the final manuscript.

## Funding

This research was supported, in part, by Canadian Research Chair in Economics of Infectious Diseases at the University Health Network in Toronto, Ontario held by BS (CRC-950-232429).

## Conflict of interest

The authors declare that the research was conducted in the absence of any commercial or financial relationships that could be construed as a potential conflict of interest.

## Publisher's note

All claims expressed in this article are solely those of the authors and do not necessarily represent those of their affiliated organizations, or those of the publisher, the editors and the reviewers. Any product that may be evaluated in this article, or claim that may be made by its manufacturer, is not guaranteed or endorsed by the publisher.

## Abbreviations

CVD: Cardiovascular disease
EQ-5D-5L: EuroQoL life questionnaries five dimensions with five levels
EQ-VAS: EuroQoL Visual Analog scale
HRQoL: Health-Related Quality of Life
IQR: interquartile range
LMICs: Low-middle and low-income countries
MGL: Morisky Green-Levine questionnaire
SD: Standard deviation
TSQM: Treatment Satisfaction Questionnaire for Medication.
